# Supplementary material for: Molecular Analysis of SARS-CoV-2 Lineages in Armenia
Source: Viruses. 2022 May 17;14(5):1074. doi: 10.3390/v14051074 (PMC9142918; doi:10.3390/v14051074)
Supplement: Supplementary file 1 [file viruses-14-01074-s001.zip › Supplementary Fugures.pdf]

Molecular Analysis of SARS-CoV-2 Genetic Lineages in Armenia

Supplementary data

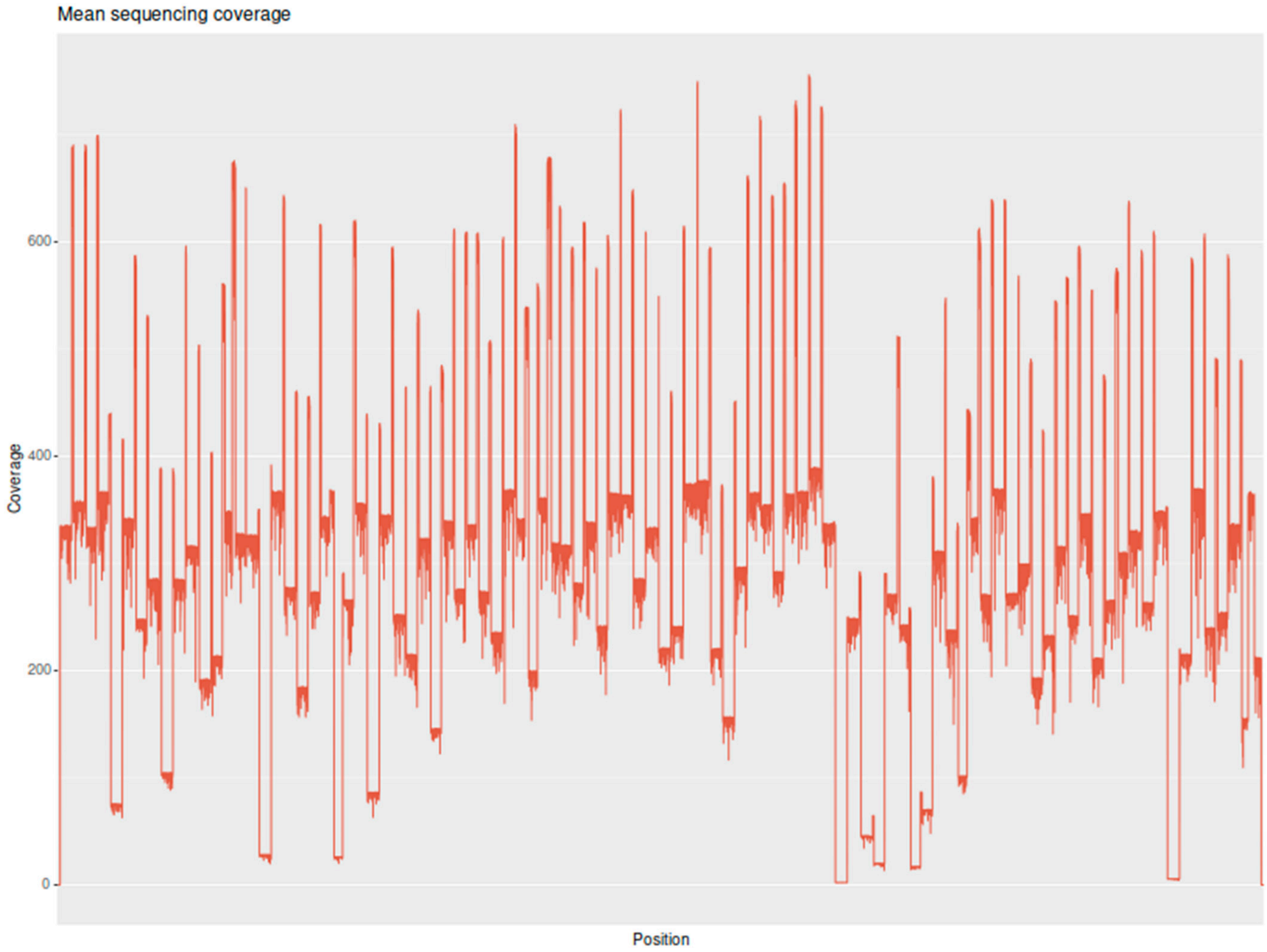

Figure S1. The average sequencing coverage for nanopore sequencing runs. The order of the positions corresponds to the reference genome (MN908947.3).

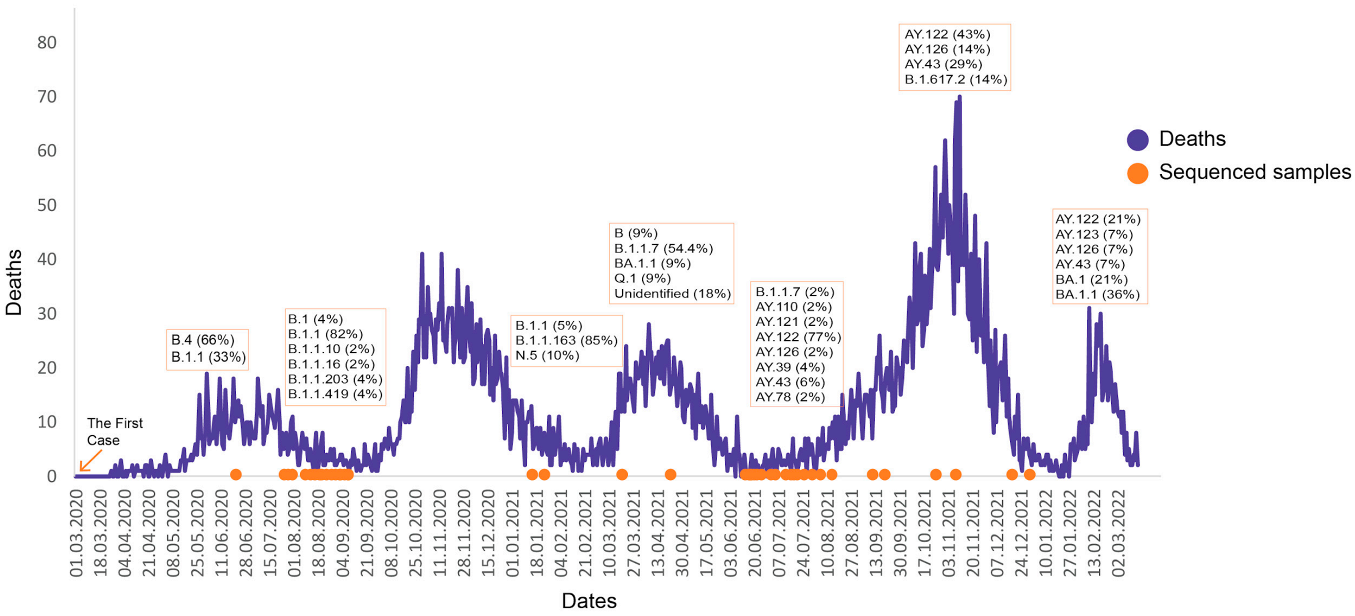

Figure S2. Daily reported deaths from COVID-19 in Armenia, sampling dates, and clade distribution of sequenced samples.

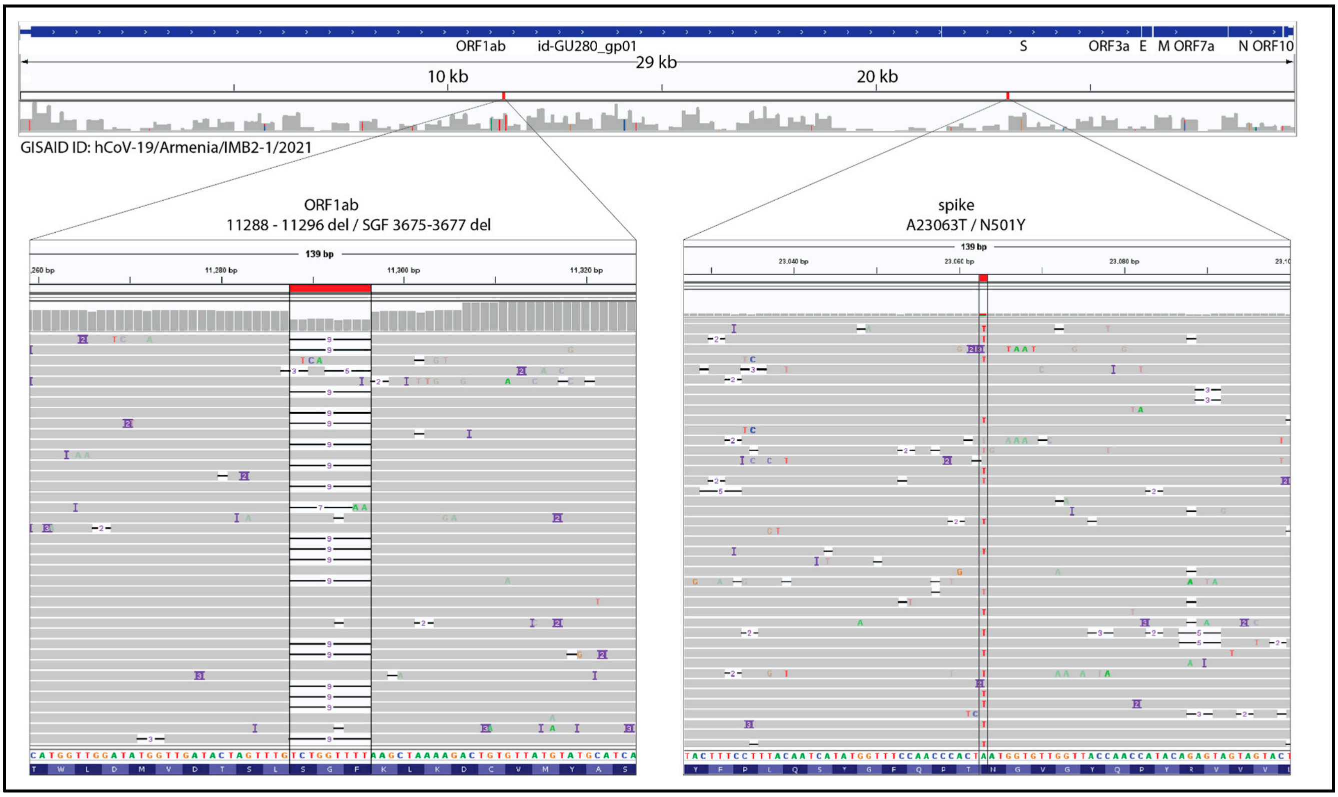

Figure S3. B.1.1.7 characteristic mutations in raw nanopore reads of IMB2-1/2021 isolate.

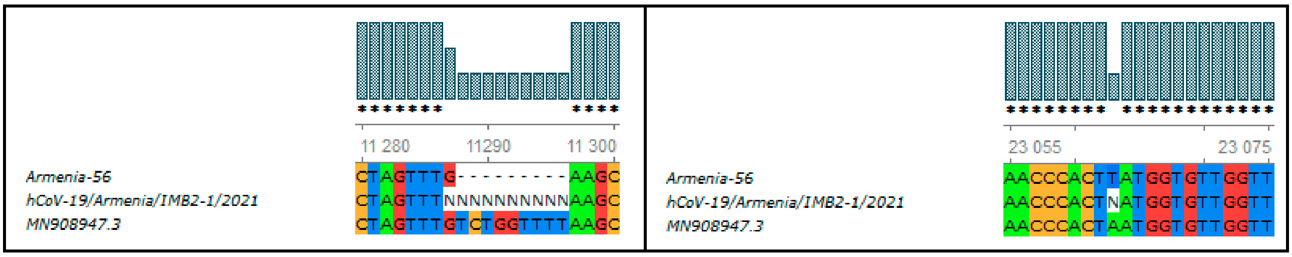

Figure S4. B.1.1.7 characteristic mutations in nanopore and Illumina consensus sequences for IMB2-1/2021 isolate. Armenia-56 - Illumina sequencing, hCov-19/Armenia/IMB2-1/2021 - nanopore sequencing, MN908947.3 - reference genome. Left panel: 20I (Alpha, V1) characteristic deletion S:H69-; right panel: 20I (Alpha, V1) characteristic mutation S:N501Y. It can be noted that in the nanopore consensus sequence mutation positions were marked with ambiguous nucleotides, while they were correctly called in the Illumina consensus sequence.
